# Involvement of TREK-1 Channel in Cell Viability of H9c2 Rat Cardiomyoblasts Affected by Bupivacaine and Lipid Emulsion

**DOI:** 10.3390/cells8050454

**Published:** 2019-05-14

**Authors:** Jun Ho Yang, Adrian S. Siregar, Eun-Jin Kim, Marie Merci Nyiramana, Eui-Jung Shin, Jaehee Han, Ju-Tae Sohn, Jong Woo Kim, Dawon Kang

**Affiliations:** 1 Departments of Medicine and Thoracic and Cardiovascular Surgery, College of Medicine, Gyeongsang National University, Jinju 52727, Korea; junhoyah@hanmail.net; 2 Gyeongsang National University Changwon Hospital, Changwon 51472, Korea; 3 Department of Physiology, College of Medicine and Institute of Health Sciences, Gyeongsang National University, Jinju 52727, Korea; adriansiregar46@gmail.com (A.S.S.); eunjin1981@hanmail.net (E.-J.K.); mariemerci0222@gmail.com (M.M.N.); eui-jung@naver.com (E.-J.S.); jheehan@gnu.ac.kr (J.H.); 4 Department of Convergence Medical Science, Gyeongsang National University, Jinju 52727, Korea; 5 Department of Anesthesiology and Pain Medicine, College of Medicine, Gyeongsang National University, Jinju 52727 Korea; jtsohn@gnu.ac.kr

**Keywords:** bupivacaine, cardiomyoblast, lipid emulsion, membrane potential, TREK-1

## Abstract

Lipid emulsion (LE) therapy has been used to reduce overdose of bupivacaine (BPV)-induced cardiotoxicity. The TWIK-related potassium channel-1 (TREK-1) is inhibited by BPV and activated by polyunsaturated fatty acids, which are the main component in LE. These pharmacological properties inspired us to investigate whether the TREK-1 channel is associated with cell viability of H9c2 cardiomyoblasts affected by BPV and LE. Consistent with previous studies, BPV-induced cell death was reduced by LE treatment. The reduction in the TREK-1 expression level by BPV was alleviated by LE. The BPV cytotoxicity highly decreased in TREK-1 overexpressed cells but was the opposite in TREK-1 knocked-down cells. TREK-1 channel activators and inhibitors increased and decreased cell viability, respectively. BPV-induced depolarization of the plasma and mitochondrial membrane potential and increase in intracellular Ca^2+^ level were blocked by LE treatment. BPV-induced depolarization of membrane potential was reduced in TREK-1 overexpressed cells, indicating that TREK-1 channels mediate setting the resting membrane potentials as a background K^+^ channel in H9c2 cells. These results show that TREK-1 activity is involved in the BPV cytotoxicity and the antagonistic effect of LE in H9c2 cells and suggest that TREK-1 could be a target for action of BPV and LE.

## 1. Introduction

Bupivacaine, a local anesthetic of the amide group frequently used for spinal and epidural anesthesia during labor as well as for postoperative pain management, is more cardiotoxic compared with other local anesthetics [1]. Bupivacaine may cause adverse drug reactions in the cardiovascular system, such as a negative inotropic effect, decrease in blood pressure and heart rate, alteration in electrical excitability, depressed contractility, and increased afterload through high plasma concentrations, lost Na^+^ channel binding properties, and block of other channels [2,3]. Bupivacaine affects many types of ion channels [4]. An overdose of bupivacaine induces vasodilation of blood vessels and circulatory collapse due to its cardiotoxicity. Attempts have been made to reduce these bupivacaine-induced systemic toxicities via lipid emulsion (LE) therapy. 

LE has been used as a source of energy and parenteral nutrition for patients since 1962 [5]. LE is well-known as a nonspecific antidote in medicine, in particular for bupivacaine-induced cardiotoxicity [6]. LE accelerates recovery from bupivacaine toxicity in isolated rat heart [7], inhibits apoptosis and cardiotoxicity induced by bupivacaine or doxorubicin in rat cardiomyoblasts [8,9], reduces bupivacaine content, and increases cardiac bupivacaine washout [7,10]. The protective mechanisms of LE on bupivacaine-induced cardiotoxicity have been generally thought to be lipid sink, lipid shuttle, fatty acid supply, reversal of mitochondrial dysfunction and cardiac Na^+^ channel blockade, inhibition of nitric oxide release, and inotropic effects [11]. In addition to the above mechanisms, LE modulates ion channels like bupivacaine.

A background K^+^ channel TWIK-related potassium channel (TREK)-1, a member of two-pore domain K^+^ (K_2P_) channel, contributes to set the resting membrane potential in many types of cells including heart cells. The TREK-1 channel is expressed in rodent and human heart cells [12,13]. The TREK-1 channel is activated by low intracellular pH, high temperature, stretch, and free fatty acids [14,15,16], and it is inhibited by bupivacaine treatment in a reversible manner [17,18,19]. Free fatty acids are the main component in LE. The TREK-1 channel is thought to be closely related to the protective role of LE in bupivacaine-induced cytotoxicity based on its pharmacological properties. These pharmacological properties of TREK-1 channel could enhance the antagonistic effect of LE on bupivacaine-induced cytotoxicity in H9c2 cells. However, little is known about the role of the TREK-1 channel in the action of bupivacaine and LE. This study was performed to investigate the role of TREK-1 channel in the action of LE and bupivacaine in H9c2 rat cardiomyoblast cells.

## 2. Materials and Methods

### 2.1. Chemicals

Unless otherwise stated, all chemicals were purchased from Sigma Aldrich (St Louis, MO, USA). Stock solution of bupivacaine (100 mM; TCI, Tokyo, Japan), BaCl_2_ (1 M), EGTA (ethylene glycol-bis(oxyethylenenitrilo)tetraacetic acid, 0.5 M), fluoxetine (10 mM), and tetraethylammonium (TEA, 1 M) were prepared in distilled water. Paroxetine (20 mM) and tetrodotoxin (TTX, 2 mM; Alomone Labs, Jerusalem, Israel) were prepared in dimethyl sulfoxide (DMSO) and acidic buffer (pH 4.8), respectively. Spadin (1 mg/mL, Lot #K190483) was synthesized by the ANYGEN (Gwangju, South Korea) and was dissolved in DMSO. LE (Intralipid 20%; Fresenius Kabi AB, Uppsala, Sweden) was diluted in culture medium to the working concentration. Arachidonic acid (liquid, 3.03 M) was serially diluted in the order of DMSO and culture medium to the working concentration.

### 2.2. Cell Culture

Human embryonic kidney cell line HEK-293 cells were obtained from the Korean Cell Line Bank (Seoul, Korea). Rat cardiomyoblast cell line H9c2 and HEK-293 cells were cultured in Dulbecco’s modified Eagle’s medium (DMEM; Gibco/Life technologies, Grand Island, NY, USA) supplemented with 10% fetal bovine serum (FBS; Gibco), 100 U/mL penicillin (Gibco), and 100 mg/mL streptomycin (Gibco). The cells were incubated at 37 °C in 95% air and 5% CO_2_ gas mixture, and the medium was replaced every other day. For extracellular Ca^2+^ free and Na^+^ channel blockade, CaCl_2_ was omitted, and 1 μM TTX (Alomone Labs) was added to the culture medium.

### 2.3. Cell Viability Assay 

Cell viability of H9c2 was determined using a 3-(4,5-dimethylthiazole-2-yl)-2,5-diphenyl tetrazolium bromide (MTT) reagent (5 mg/mL in phosphate buffered saline (PBS), Duchefa Biochemie, Haarlem, The Netherlands). H9c2 cells were seeded at 5 × 10^3^ cells/well (100 μL) in 96-well plates, and the cells were cultured for 24 h before treatment with chemicals. Chemicals were treated for 24 h. MTT solution (10 μL of 5 mg/mL) was added to each well and incubated for 2 h at 37 °C in the dark. The supernatants were aspirated and then the formazan crystals in each well were dissolved in 100 μL DMSO for 10 min at room temperature by shaking. The absorbance was measured at 570 nm using VERSAmax™ microplate reader (Molecular Devices, San Jose, CA, USA).

### 2.4. Transfection

Transfection was performed with Magnetofection™ reagent according to the manufacturer’s protocol (chemicell GmbH, Berlin, Germany). Briefly, HEK-293 and H9c2 cells were cultured 24 h prior to transfection at a density 2 × 10^5^ cells per 60-mm dish in DMEM containing 10% FBS. H9c2 cells were transfected with DNA fragments encoding rat TREK-1 (AF385402) in pcDNA3.1 or TREK-1 small interfering RNA (siRNA) using Magnetofection reagents (chemicell GmbH) and Opti-MEM I Reduced Serum Medium (Opti-MEM, Gibco). For the electrophysiological study, the cells were grown on round cover glass coated with poly-l-lysin in a 35-mm dish then were co-transfected with rat TREK-1 plasmid DNA and green fluorescent protein (GFP) in pcDNA3.1 using SuperFect Transfection Reagent (Qiagen, Hilden, Germany). Green fluorescence from cells expressing GFP was detected with the aid of a Zeiss microscope (Jena, Germany) equipped with a mercury lamp light source. Cells were used 2–3 days after transfection.

For gene silencing with small interfering RNA (siRNA), H9c2 cells were transfected with 75 nM of scrambled siRNA (negative control, ON-TARGET Non-Targeting Pool; Dharmacon, Lafayette, CO, USA) or TREK-1 ON-TARGETplus SMARTpool siRNA (Dharmacon) in serum-free medium using DharmaFECT transfection reagent (Dharmacon). After incubation for 6 h, cells were changed with fresh medium and grown for an additional day. The effects of gene silencing were determined by reverse transcriptase polymerase chain reaction (RT-PCR) and Western blot analyses.

### 2.5. Live/Dead Cell Staining

Live/dead cell staining was performed using calcein-AM (Thermo Fisher Scientific, Eugene, OR, USA) and propidium iodide (PI). Viable cells and disordered areas of dead cell membrane are stained with Calcein-AM and PI and appear green and red, respectively. H9c2 cells (5 × 10^3^ cells/100 μL) were cultured on glass-bottom culture dish (SPL, Pocheon, Korea) for 24 h. The cells were pretreated with LE for 1 h and then stimulated with bupivacaine for 24 h. The cells were washed twice with Opti-MEM and then stained with 3 μM calcein-AM and 3 μg/mL PI for 25 min at room temperature. The stained cells were washed and observed using a confocal laser scanning microscope (Olympus, Tokyo, Japan) with filter sets for Texas Red and fluorescein isothiocyanate (FITC).

### 2.6. RT-PCR and Real-Time PCR

Total RNA isolated from H9c2 cells was used for synthesis of the first-strand cDNA using reverse transcriptase kit (DiaStartTM RT kit; SolGent, Daejeon, Korea). The first-strand cDNA, specific primers for rat TREK-1, and Taq polymerase (G-Taq, Cosmo Genetech, Seoul, Korea) were used for PCR amplification. The sequences of the PCR primer pairs were listed in Table 1. Glyceraldehydes-3-phosphate dehydrogenase (GAPDH) was used as a loading control. The PCR conditions included initial denaturation at 94 °C for 5 min, then 30 cycles at 94 °C for 30 s, 55 °C for 30 s, and 72 °C for 30 s, and a final extension step at 72 °C for 10 min. The PCR products were electrophoresed on 1.5% (*w/v*) agarose gel to verify the product size. The images of DNA fragments were directly captured with a gel imaging system with a digital camera (Canon, Tokyo, Japan) and ultraviolet (UV) transilluminator (Vilber Lourmat, Marne La Vallee, France). The expected bands were extracted and directly sequenced with an ABI PRISM^®^ 3100-Avant Genetic Analyzer (Applied Biosystems, Foster City, CA, USA).

Changes in TREK-1 expression identified by RT-PCR were quantified using real-time PCR with the TOPreal™ SYBR Green 2× PreMix (Enzynomics, Daejeon, South Korea) and Lighter Cycler^®^ 480 II/96 system (Roche Diagnostics Ltd., Rotkreuz, Switzerland). The PCR conditions for real-time PCR were as follows: initial denaturation at 95 °C for 5 min, 45 cycles for denaturation at 95 °C for 30 s, annealing at 60 °C for 30 s, and extension at 72 °C for 30 s. The 2^-ΔΔC^_T_ method was used to calculate the relative levels of TREK-1 mRNA [20]. TREK-1 mRNA expression was normalized to reference genes, *GAPDH,* and ribosomal protein S12 (*RPS12*), which show stable expression in H9c2 cells [21].

### 2.7. Western Blot Analysis

H9c2 cells (2 × 10^5^ cells/60-mm dish) were treated with chemicals for 24 h. Total protein was isolated from the cells using the RIPA lysis and extraction buffer (Thermo Fisher Scientific) containing 25 mM Tris-HCl (pH 7.4), 150 mM NaCl, 1% NP-40, 1% deoxycholate, 0.1% sodium dodecyl sulfate (SDS), and 1× protease inhibitor cocktail (Roche Diagnostics, Indianapolis, IN, USA). The cell lysates were incubated for 30 min on ice with intermittent vortexing and were clarified by centrifugation at 16,609× *g* (13,000 rpm, Hanil, Incheon, Korea) at 4 °C for 20 min. After centrifugation, the supernatant was separated and stored at −70 °C until use.

Protein concentration in cell lysates was quantified using a Pierce bicinchoninic acid (BCA) protein assay kit (Thermo Fisher Scientific). Equal amounts of proteins mixed with 1× loading buffer among groups were separated on 12% sodium dodecyl sulfate (SDS)-polyacrylamide gel, and the gel was blotted onto a polyvinylidene difluoride (PVDF, Millipore, Billerica, MA, USA) membrane for 15 min using a semi-dry transfer (Bio-Rad, Hercules, CA, USA). Membranes were blocked with 5% (*w*/*v*) fat-free dry milk in tris buffered saline with tween20 (TBST; 20 mM Tris HCl (pH 8), 137 mM NaCl, and 0.2% Tween-20) at room temperature for 60 min and then incubated with polyclonal anti-TREK-1 (1:1000 dilution, Alomone Labs, batch # AN-02), monoclonal anti-β-actin antibody (1:5000 dilution, batch # 026M4780V), or monoclonal anti-α-tubulin (1:5000 dilution, batch # 102M47773V) at 4 °C overnight. The primary antibody incubation was followed by incubation with a secondary horseradish peroxidase (HRP)-conjugated anti-rabbit or anti-mouse antibody at 1:10000 (Assay Designs, Ann Arbor, MI, USA). Immuno-positive bands were visualized by an enhanced chemiluminescence (EzWestLumi plus; ATTO Gentaur, Tokyo, Japan) according to the manufacturer’s instructions. The relative protein level was calculated using β-actin or α-tubulin as a loading control.

### 2.8. Immunocytochemistry

H9c2 cells were grown on round cover glass coated with poly-l-lysine for 24 h at 2.5 ×10^4^ cells/well (500 μL) in 24-well plates. The cells were fixed with 4% paraformaldehyde in 0.1 M PBS for 30 min, washed three times with 1× PBS, and preincubated in a blocking buffer containing 1% normal goat serum and 0.1% Triton X-100 for 2 h at room temperature under gentle rotation. The cells were incubated overnight in affinity-purified polyclonal anti-TREK-1 antibody (1:200 dilution, Alomone Labs) at 4 °C. After triple washes in PBS, the cells were incubated in the dark for 1.5 h with FITC-conjugated anti-rabbit immunoglobin G (IgG) fluorescent secondary antibody diluted 1:500 in PBS. The cells were washed three times and stained with 4′,6′-diamidino-2-phenylindole (DAPI) for nuclei staining. The stained cells were wet-mounted on glass slides and observed using a confocal laser scanning microscope (Olympus). The negative control was analyzed by omitting the primary antibody.

### 2.9. Electrophysiological Studies

Electrophysiological recording was performed using a patch clamp amplifier (Axopatch 200, Axon Instruments, Union City, CA, USA). Single-channel currents were filtered at 2 kHz using an 8-pole Bessel filter (−3 dB; Frequency Devices, Haverhill, MA, USA) and transferred to a computer (Samsung, Suwon, Korea) using the Digidata 1320 interface (Axon Instruments) at a sampling rate of 20 kHz. Threshold detection of channel openings was set at 50%. In experiments using cell-attached and excised patches, the pipette and bath solutions contained (in mM): 150 KCl, 1 MgCl_2_, 5 EGTA, and 10 HEPES (pH 7.3). For whole-cell currents, bath solution contained (in mM): 135 NaCl, 5 KCl, 1 CaCl_2_, 1 MgCl_2_, 5 glucose, and 10 HEPES, and pipette solution contained (in mM): 150 KCl, 1 MgCl_2_, 5 EGTA, and 10 HEPES (pH 7.3). All solutions were prepared with Milli-Q water (18.2 MΩ-cm at 25 °C). Whole-cell current was recorded in response to a voltage ramp (−120 to +60 mV; 1 s duration) and a step (−120 to +60 mV; 2 s duration) from a holding potential of −80 mV. The currents measured at +60 mV were analyzed. Single-channel and whole-cell currents were analyzed with the pCLAMP program (version 10, Axon). Channel activity (NP_o_, where N is the number of channels in the patch and P_o_ is the probability of a channel being open) was determined from ~1 to 2 min of current recording. All experiments were performed at ~25 °C. For recording background K^+^ currents in H9c2 cells, 4-Aminopyridine (4-AP, 1 mM), BaCl_2_ (1 mM), and TEA (1 mM) were added to bath solution to rule out the involvement of other K^+^ channels.

Membrane potential was recorded in the same pipette and bath solutions as those for whole-cell currents under current-clamp mode (I *=* 0), which is useful for recording the membrane potential by injecting current into a cell through the recording electrode.

### 2.10. Measurement of Intracellular Ca^2+^ Concentration

The intracellular Ca^2+^ was measured using a confocal laser scanning microscope equipped with a fluorescence system (IX70 Fluoview, Olympus). H9c2 cells cultured on a glass-bottom culture dish (SPL) were incubated with 5 μM Fluo-3AM in serum free DMEM media for 30 min and washed three times with 1× PBS. Each fluorescent image was scanned every 5 s at 488 nm on an excitation argon laser and 530 nm long pass emission filters. All scanned images were processed to analyze changes in intracellular Ca^2+^ concentration [Ca^2+^]_i_ at the single-cell level. In each cell studied, the changes in [Ca^2+^]_i_ were calculated as fluorescence intensity (F) divided by the basal fluorescence intensity before treatment (F_0_) to control for variations in basal fluorescence (F/F_0_). Net changes in F are represented as (F_max_ − F_0_)/F_0_, where F_max_ is the maximum level of fluorescence intensity, which occurred after the addition of chemicals. The changes in [Ca^2+^]_i_ were measured for 8 min after treatment with chemicals, because the change in [Ca^2+^]_i_ is an immediate reaction in response to chemicals.

### 2.11. Measurement of Plasma and Mitochondrial Membrane Potentials Using Dye

The plasma membrane potential (PMP) was measured with the FluoVolt™ membrane potential kit (Thermo Fisher Scientific) using the IX70 Fluoview (Olympus). The FluoVolt™ membrane potential dye represents fast and slow response membrane potential changes. Cells grown on glass-bottom culture dishes (SPL) were incubated with the FluoVolt™ Loading Solution containing 1× FluoVolt™ dye and PowerLoad™ concentrate in a physiological solution for 25 min at room temperature. The cells were washed three times with the physiological solution. The glass-bottom culture dish containing cells were placed on a confocal laser scanning microscope, and the cells were scanned with a standard FITC filter set. Each fluorescent image was scanned every 5 s at 488 nm on an excitation argon laser and 530 nm long pass emission filters. Time-lapse images were processed to analyze changes in PMP at a single-cell level. Net changes in F are represented as (F_max (min)_ − F_0_)/F_0_. F_max_ or F_min_ is the maximum or minimum level of fluorescence intensity, which occurred after the addition of chemicals, respectively. The physiological solution contained (in mM): 135 NaCl, 5 KCl, 1 CaCl_2_, 1 MgCl_2_, 5 glucose, and 10 HEPES (pH 7.3, 300 mOsm/L).

Mitochondrial membrane potential (MMP) changes were determined by JC-1 mitochondrial membrane potential detection kit (Biotium Inc. Hayward, CA, USA) according to the manufacturer’s protocol. Briefly, H9c2 cells (2 × 10^5^ cells/60-mm dish) grown on glass-bottom culture dishes were treated with bupivacaine and/or LE for 24 h, stained with 1× JC-1 reagent at 37 °C for 15 min, and resuspended with 1× PBS. Changes in MMP were measured at the single cell level by fluorescence image analysis. Mitochondrial function was usually monitored with changes in the fluorescence intensity ratio (red/green).

### 2.12. Measurement of Intracellular K^+^ Concentration

The intracellular K^+^ concentration ([K^+^]_i_) was measured with PBFI K^+^-sensitive dye (Thermo Fisher Scientific) according to the manufacturer’s protocol. Briefly, PBFI (5 μM) was treated 30 min before the measurement of [K^+^]_i_ in the H9c2 cells treated with bupivacaine and/or LE for 24 h. The PBFI solution was removed and washed three times with 1× PBS. Each fluorescent image was scanned at excitation (340/380 nm) and emission (500 nm) wavelengths. The images were processed to analyze changes in [K^+^]_i_ at the single-cell level.

### 2.13. Data Analysis and Statistics

The bands obtained from the RT-PCR and Western blot tests were quantified by using Sigma Gel image analysis software (version 1.0, Jandel Scientific, San Rafael, CA, USA) or Quantity One software (version 4.6.3) attached to a GS-800 calibrated densitometer (Bio-Rad). Fluoview (FV1000, Ver. 1.5, Olympus) was used to analyze immunofluorescence data. Data are represented as mean ± SD. Significant differences between groups were analyzed by using one-way analysis of variance (ANOVA) with post-hoc comparisons using Tukey’s test (OriginPro 8, Northampton, MA, USA). A *p* < 0.05 was considered as the criterion for statistical significance.

## 3. Results

### 3.1. Reduction in Bupivacaine-Induced Cell Death in H9c2 Cells by LE

**Figure 1:**
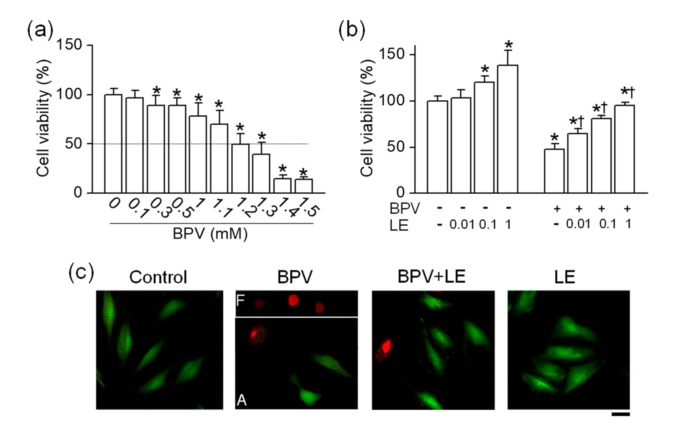
Effect of lipid emulsion (LE) on bupivacaine (BVP)-induced cell death in H9c2 cells. (**a**) Concentration-dependent effect of bupivacaine on cell viability. * *p* < 0.05 compared to control without bupivacaine treatment. (**b**) Effect of LE alone and combination of bupivacaine and LE on cell viability. * *p* < 0.05 compared to no treatment. ^†^ *p* < 0.05 compared with bupivacaine only treatment. (c) Live and dead cells displayed in green (Carcein) and red (propidium iodide, PI), respectively. Each bar represents the mean ± SD of three independent experiments. The scale bar represents 50 μm. BPV and LE represent bupivacaine and LE, respectively. F and A in bupivacaine treatment represent floated and attached cells, respectively.

To identify the effects of bupivacaine and LE on the viability of H9c2 cells, H9c2 cells were treated with various concentrations of bupivacaine and/or LE for 24 h, and the cell viability was determined by MTT assay. Bupivacaine (0.1 to 1.5 mM) treatment decreased the cell viability in a concentration-dependent manner (Figure 1a). Bupivacaine significantly induced cell death from the concentration of 300 μM under the current experimental conditions, and the half maximal inhibitory concentration (IC_50_) of bupivacaine was 1.2 ± 0.2 mM (*n* = 6, *p* < 0.05, Figure 1a). The cells were treated with different concentrations of LE exposed to 1.2 mM bupivacaine to identify its protective effect on bupivacaine-induced cell death. LE treatment alone significantly increased the cell viability in a concentration-dependent manner, and combined treatment with bupivacaine and LE (0.01 to 1%) significantly decreased bupivacaine-induced cell death compared with bupivacaine treatment alone (*n* = 6, *p* < 0.05, Figure 1b). Additional study was performed with 1.2 mM bupivacaine and 0.1% LE. Higher PI-positive dead cells were seen in the bupivacaine only treatment, but the number of PI-stained cells decreased in the cotreatment with bupivacaine and LE (*n* = 3, Figure 1c). The images of floated and attached cells were separately captured because of the focus difference. In the bupivacaine only treatment, PI-positive cells were markedly detected in floated cells.

### 3.2. Antagonistic Effect of LE on Bupivacaine-Induced Decrease in TREK-1 Expression Level 

**Figure 2:**
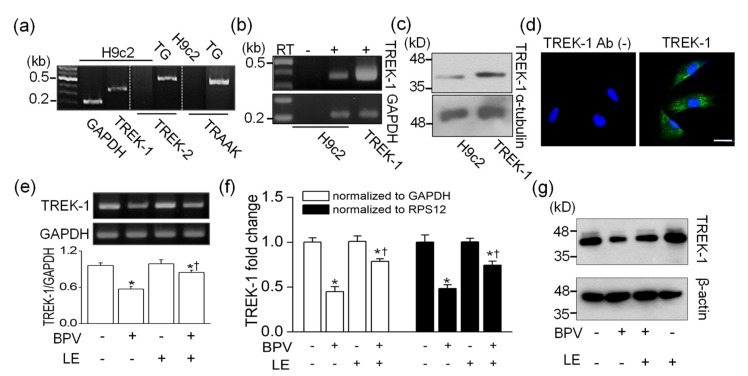
Changes in TWIK-related potassium channel (TREK)-1 expression level in H9c2 cells by bupivacaine and LE. (**a**) No detection of TREK-2 and TWIK-related arachidonic acid-activated potassium channel (TRAAK) expression. Trigeminal ganglion (TG) was used as a positive control for expression of TREK-2 and TRAAK. (**b**) TREK-1 mRNA expression. Rat TREK-1 transfected HEK-293 cells and glyceraldehydes-3-phosphate dehydrogenase (GAPDH) were used as a positive control and a loading control, respectively. RT represents reverse transcriptase. The signs of + and – represent treatment condition with and without bupivacaine and/or LE, respectively. (**c**) TREK-1 protein expression. Protein obtained from rat TREK-1 overexpressed cells was used as a positive control, and α-tubulin was used as a loading control. (**d**) Localization of TREK-1 protein. Green and blue indicate TREK-1 expression and nucleus stained with DAPI, respectively. The scale bar represents 30 µm. Changes in TREK-1 mRNA expression level detected by (**e**) semi-quantitative PCR and (**f**) real-time PCR. (**g**) Changes in TREK-1 protein level by bupivacaine and LE. Cells were treated with bupivacaine and/or LE for 24 h. We analyzed 1 μg of cDNA and 30 μg of protein per lane by semi- and real-time quantitative PCR and immunoblotting, respectively. *GAPDH* and *RPS12* were used as reference genes for real-time PCR. β-actin was used as a loading control for quantification of protein level. Each bar represents the mean ± SD of four independent experiments. * *p* < 0.05 compared to control. ^†^ *p* < 0.05 compared to bupivacaine treatment.

The expression of TREK-2 and TRAAK, which are included in the same subfamily as TREK-1 in K_2P_ channel family, was checked in H9c2 cells. Despite several attempts, TREK-2 and TRAAK mRNAs were not detected. RT-PCR data showed that TREK-1 mRNA was expressed in H9c2 cells (*n* = 4, Figure 2a). The expression of TREK-1 in H9c2 cells was investigated in detail using RT-PCR, Western blotting, and immunocytochemistry. The mRNA and protein obtained from HEK-293 cells transfected with rat TREK-1 were used as positive controls for RT-PCR and Western blotting, respectively. The TREK-1 mRNA was not detected in H9c2 cDNA synthesized without reverse transcriptase, indicating that genomic DNA was not contaminated in the sample (*n* = 4, Figure 2b). The Western blotting assay also showed expression of TREK-1 protein in H9c2 cells (*n* = 4, Figure 2c). The TREK-1 protein was localized at intracellular compartments and plasma membrane of H9c2 cells, except nucleus, whereas only DAPI stains were seen in negative control with no anti-TREK-1 antibody treatment (*n* = 6, Figure 2d). 

To identify whether bupivacaine and/or LE affects TREK-1 expression level, semi-quantitative RT-PCR, real-time PCR, and Western blotting were performed in H9c2 cells treated with the bupivacaine and/or LE for 24 h. Bupivacaine treatment significantly decreased TREK-1 mRNA expression level, whereas cotreatment with bupivacaine and LE significantly recovered TREK-1 expression level reduced by bupivacaine to near to the control level (*n* = 4, *p* < 0.05). LE treatment alone did not affect TREK-1 expression level (Figure 2e). The changes in TREK-1 expression level were quantified by real-time PCR. The mRNA expression level of TREK-1 was reduced by bupivacaine treatment, and the bupivacaine-induced reduction was recovered by cotreatment with bupivacaine and LE (*n* = 4, *p* < 0.05, Figure 2f). TREK-1 protein expression was also decreased by bupivacaine treatment, and the reduced expression was recovered by combination of bupivacaine and LE (*n* = 3, Figure 2g).

### 3.3. Cell Viability Changed in TREK-1 Manipulated H9c2 Cells 

**Figure 3:**
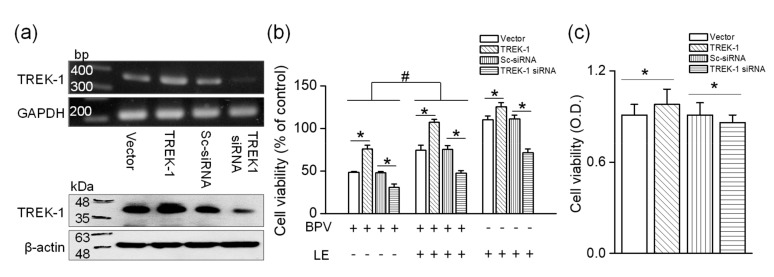
Effect of bupivacaine and/or LE on TREK-1 manipulated H9c2 cells. (**a**) Efficiency of TREK-1 or TREK-1 small interfering RNA (siRNA) transfection on H9c2 cells. pcDNA3.1 (vector), TREK-1 in pcDNA3.1 (TREK-1), scrambled siRNA (Sc-siRNA), or TREK-1 siRNA was transfected into H9c2 cells. PCR (upper panel) and immunoblotting (lower panel) assays were performed on day 2 after transfection. The TREK-1 siRNA transfection was performed twice (36 h apart). We analyzed 1 μg of cDNA and 30 μg of protein per lane by semi-quantitative PCR and immunoblotting, respectively. GAPDH and β-actin were used as loading controls for PCR and immunoblotting, respectively. (**b**) Altered cell viability in TREK-1 overexpressed and knocked-down cells by treatment with bupivacaine and/or LE. The relative cell viability was calculated by dividing each control, which is a group only transfected with vector, TREK-1, scrambled siRNA, or TREK-1 siRNA without bupivacaine and/or LE treatment. The signs of + and – represent treatment condition with and without chemicals, respectively. Each bar represents the mean ± SD of three independent experiments. * *p* < 0.05 compared to each corresponding control. ^#^ *p* < 0.05 compared to bupivacaine treatment alone and bupivacaine and LE cotreatment. BPV and LE represent bupivacaine and lipid emulsion, respectively. (**c**) Significant effect of TREK-1 expression level on viability of H9c2 cells. The cells were transfected with vector, TREK-1, scrambled siRNA, or TREK-1 siRNA, and the cell viability was evaluated on day 2 after transfection. Data were analyzed by using Mann–Whitney test: * *p* < 0.05 compared to each corresponding control. OD represents optical density.

As shown in Figure 2, bupivacaine and LE regulated the TREK-1 expression level. To identify whether TREK-1 expression level affects the regulatory effects of bupivacaine and/or LE on cell viability, the TREK-1 manipulated cells were treated with bupivacaine and/or LE. The effects were compared to a corresponding control. In TREK-1 manipulated cells, the transfection efficiency was evaluated by RT-PCR and Western blotting assays. H9c2 cells transfected with TREK-1 showed high mRNA/protein expression levels compared to vector transfected cells, whereas TREK-1 knocked-down cells showed low expression levels compared to scrambled siRNA transfected cells (*n = 3*, Figure 3a). Bupivacaine cytotoxicity was significantly lower in TREK-1 overexpressed cells than that in vector transfected cells. However, bupivacaine induced higher cell death in TREK-1 knocked-down cells than that in scrambled siRNA transfected cells (*n* = 6, *p < 0.05*, Figure 3b). Combination of bupivacaine and LE completely recovered the cell death induced by bupivacaine in TREK-1 overexpressed cells, but not in TREK-1 siRNA transfected cells. The percentage of cell viability protected by LE ranged from 42 to 58% in all groups. LE alone treatment increased cell viability in TREK-1 overexpressed cells, whereas it decreased the cell viability by 29.8 ± 4.3% in TREK-1 knocked-down cells compared with each corresponding control (*n* = 6, *p* < 0.05, Figure 3b). Only the expression level of TREK-1 affected significantly the cell viability (*n* = 28, *p* < 0.05, Figure 3c). TREK-1 overexpressed cells and TREK-1 siRNA transfected cells showed an increase and a decrease in cell viability, respectively, compared to each corresponding control. 

### 3.4. Functional Expression of TREK-1-Like Channel in H9c2 Cells

**Figure 4:**
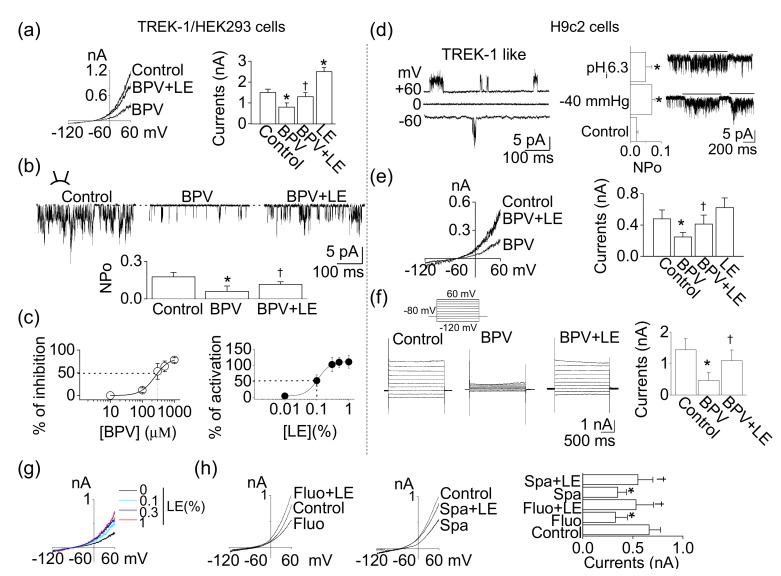
Functional expression of TREK-1 in H9c2 cells. (**a**) Effects of bupivacaine and/or LE on TREK-1 overexpressed HEK-293 cells. Whole-cell currents were recorded in 5mM KCl, and the current levels at +60 mV were determined and analyzed. (**b**) Single-channel activity of TREK-1 in response to bupivacaine and/or LE under inside-out patch mode at +60 mV of pipette potential. (**c**) Inhibition and activation curves of TREK-1 channel by bupivacaine and LE, respectively. The dotted lines represent the IC_50_ and EC_50_ of bupivacaine and LE. (**d**) The single-channel recording of TREK-1-like channel in H9c2 cells. The TREK-1-like channels were activated by intracellular low pH and negative pressure. (**e**,**f**) H9c2 background whole-cell currents. Whole-cell currents were recorded from H9c2 cells in the presence of 1 mM 4-AP, 1 mM Ba^2+^, and 1 mM TEA to rule out contamination of other K^+^ channels. Cell membrane potential was held at −80 mV, and (**e**) voltage ramps or (**f**) voltage steps from –120 to +60 mV in 20 mV intervals were applied for 1 s or 2 s durations, respectively. Pipette and bath solutions contained 150 and 5 mM K^+^, respectively. (**g**) Effect of different concentrations of LE on H9c2 background K^+^ currents. (**h**) Combined effects of TREK-1 inhibitors and LE on H9c2 background K^+^ channels. Each bar (data) represents the mean ± SD of three independent experiments (*n* = 6). * *p* < 0.05 compared to the control. ^†^ *p* < 0.05 compared to the bupivacaine treatment or each corresponding control.

TREK-1 mRNA and protein were expressed in H9c2 cells (Figure 2). To identify whether the TREK-1 channel is functionally expressed in H9c2 cells, whole-cell and single-channel recordings were performed. Pharmacological properties of TREK-1 expressed in H9c2 cells were compared to those in HEK-293 cells transfected with rat TREK-1. TREK-1 was inhibited by bupivacaine, and the bupivacaine-induced inhibition was recovered by LE. LE treatment alone significantly increased TREK-1 channel activity by two-fold (*n* = 6, *p* < 0.05, Figure 4a). The excised single-channel recoding also showed a bupivacaine-induced inhibition and a blockade of bupivacaine-induced inhibition by LE (*n* = 6, *p* < 0.05, Figure 4b). The IC_50_ of bupivacaine and EC_50_ of LE for TREK-1 channel were 302.2 ± 17.6 μM and 0.09 ± 0.02%, respectively (*n* = 6, Figure 4c). 

The opening of the TREK-1-like channel was observed in cell-attached and excised patches of H9c2 cells (*n* = 6, Figure 4d). The channel activity was enhanced by application of intracellular low pH (pH_i_ 6.3) and negative pressure (−40 mmHg) (*n* = 6, *p* < 0.05). The single-channel conductance of the H9c2 TREK-1-like channel was 85.0 ± 8.5 pS and 91.5 ± 6.1 pS at −60 and +60 mV, respectively. In a physiological bath solution containing 5 mm KCl (pH_o_ 7.3), the membrane potential of cells under the whole-cell configuration was held at –80 mV, and then a ramp pulse (Figure 4e) or voltage steps (Figure 4f) were applied. Bupivacaine and LE applied to H9c2 cells showed a similar effect to those in HEK-293 cells transfected with TREK-1. Bupivacaine inhibited background K^+^ currents by 49.8 ± 6.2%, whereas the combination of bupivacaine and LE counterbalanced the inhibitory effect of bupivacaine on background K^+^ currents (*n* = 6, Figure 4e). In H9c2 cells, voltage steps elicited large current (1–2 nA at +60 mV; *n* = 6; Figure 4f). The activation of current by step depolarization was nearly instantaneous, and little inactivation was observed. Bupivacaine significantly inhibited the background K^+^ currents, and LE recovered the reduction (*p* < 0.05). The pharmacological properties of the H9c2 K^+^ channel and TREK-1 in HEK-293 cells were indistinguishable. However, the effect of the LE alone was different between them. LE slightly activated background K^+^ currents in H9c2 cells (Figure 4e). Treatment with LE increased background K^+^ currents in a concentration-dependent manner, but the increase was not that large (Figure 4g). The effect of LE was also evaluated in the presence of fluoxetine or spadin, which is known as a TREK-1 inhibitor. The background K^+^ currents in H9c2 cells was inhibited by fluoxetine (10 μM) and spadin (1 μM), and the inhibition was reduced by a combination of LE (Figure 4h). These results provide strong evidence that this H9c2 K^+^ channel is a functional correlate of TREK-1.

### 3.5. Protective Effect of TREK-1 Channel Activators on Bupivacaine-Induced Cell Death

**Figure 5:**
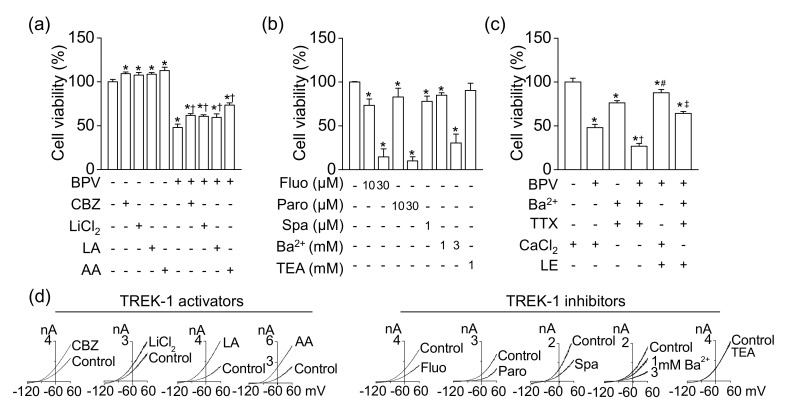
Effect of TREK-1 channel modulators on cell viability. (**a**) Increase in cell viability by TREK-1 channel activators (100 μM carbamazepine, 1 mM LiCl_2_, 5 μM linoleic acid, and 5 μM arachidonic acid). ^†^ *p* < 0.05 compared to bupivacaine only treatment. (**b**) Decrease in cell viability by TREK-1 channel inhibitors. (**c**) Effect of bupivacaine and LE on cell viability under the condition of conventional K^+^, Na^+^, and Ca^2+^ channel blockade. The cells were treated with chemicals for 24 h. ^†^ *p* < 0.05 compared to presence of Ba^2+^ and TTX and absence of CaCl_2_. ^#^ *p* < 0.05 compared to presence of bupivacaine and CaCl_2_. ^‡^ *p* < 0.05 compared to the presence of bupivacaine, Ba^2+^, and TTX and absence of CaCl_2_. (**d**) Effect of TREK-1 channel modulators on TREK-1 currents overexpressed in HEK-293 cells. Whole-cell currents were recorded in 5mM KCl, and the current levels at +60 mV were determined and analyzed. Each bar represents the mean ± SD of three independent experiments. BPV, CBZ, LA, AA, Fluo, Paro, Spa, TEA, TTX, and LE represent bupivacaine, carbamazepine, linoleic acid, arachidonic acid, fluoxetine, paroxetine, spadin, tetraethylammonium, tetrodotoxin, and LE, respectively. The + or – in CaCl_2_ represents the presence or absence of CaCl_2_ (1 mM), respectively. The signs of + and – represent treatment condition with and without chemicals, respectively. * *p* < 0.05 compared to control.

As shown in Figure 4, the TREK-1-like channel was functionally expressed in H9c2 cells. To identify the effect of TREK-1 channel modulators on bupivacaine-induced cell death, the H9c2 cells were treated with TREK-1 channel activators or inhibitors. TREK-1 channel activators, LiCl_2_ (1 mM), carbamazepine (100 μM) [22], linoleic acid (5 μM), and arachidonic acid (5 μM) increased cell viability, and bupivacaine-induced cell death was significantly reduced by TREK-1 channel activators (*n* = 6, *p* < 0.05, Figure 5a). As shown in Figure 5b, antidepressants (fluoxetine, paroxetine, and spadin), TREK-1 channel inhibitors [22,23], treatments significantly decreased cell viability (*n* = 6, *p* < 0.05). BaCl_2_ significantly induced cell death in a concentration-dependent manner (*n* = 5, *p* < 0.05). TREK-1 outward current is insensitive to TEA, a blocker for conventional K^+^ channels (K_v_ and K_ir_) [24]. TEA treatment did not affect H9c2 cell viability (Figure 5b).

To reduce the influence resulting from contamination of Na^+^, Ca^2+^, and conventional K^+^ currents, which are affected by bupivacaine, in H9c2 background K^+^ currents, the cell viability was also examined in the presence of a conventional K^+^ channel blocker (1 mM Ba^2+^), a Na^+^ channel blocker (10 nM TTX), and extracellular 0 mM CaCl_2_. The blockade showed lower cell viability than the control (Figure 5c). Bupivacaine significantly induced cell death even in the blockade of conventional K^+^ channels, Na^+^ channels, and Ca^2+^ channels, indicating that bupivacaine might produce cytotoxic effect through blocking other ion channels (*n* = 6, *p* < 0.05, Figure 5c). LE reduced bupivacaine-induced cell death. The channel could be a LE-activated background TREK-1 channel. The effects of these modulators on TREK-1 currents are displayed in Figure 5d. The effects were analyzed in TREK-1 overexpressed HEK-293 cells. Carbamazepine (100 μM), LiCl_2_ (1 mM), linoleic acid (5 μM), and arachidonic acid (5 μM) increased TREK-1 currents, but fluoxetine (10 μM), paroxetine (10 μM), spadin (1 μM), and Ba^2+^ (1 and 3 mM) inhibited the currents. TEA (1 mM) did not affect the currents.

### 3.6. LE-Induced Reversal of Membrane Potential Depolarization through TREK-1 Activation

**Figure 6:**
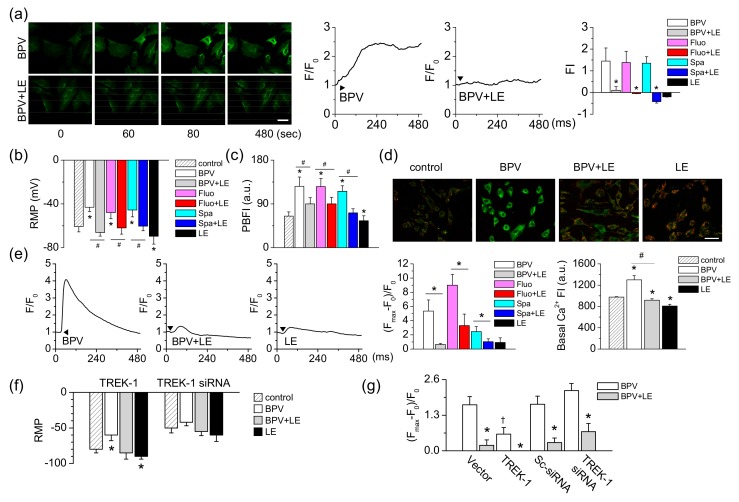
Reduction in bupivacaine-induced changes in membrane potential in TREK-1 manipulated H9c2 cells. (**a**) Changes in plasma membrane potential (PMP) by bupivacaine and LE with time. Green FluoVolt^®^ membrane labeling dye was stained and quantified the intensity using the Fluoview software program. The scale bar represents 50 μm. Typical PMP changes in response to bupivacaine and LE treatment. The bar graphs show the net changes in PMP displayed as fluorescence intensity. Arrowheads represent addition of chemicals. * *p* < 0.05 compared to each corresponding control (BPV, Fluo, Spa). (**b**) Resting membrane potential (RMP) measured under current clamp mode (I = 0). * *p* < 0.05 compared to control. ^#^ *p* < 0.05 compared to bupivacaine treatment. (**c**) Changes in [K^+^]_i_ in response to bupivacaine and/or LE. The cells were stained with PBFI K^+^ labeling dye. * *p* < 0.05 compared to the control. ^#^ *p* < 0.05 compared to bupivacaine treatment. (**d**) Changes in mitochondrial membrane potential (MMP). A specific MMP dye, JC-1, was stained to cells treated with bupivacaine and/or LE for 24 h. Green and red indicate JC-1 monomers and JC-1 aggregates, respectively. The scale bar represents 50 μm. (**e**) Typical Ca^2+^ waves in response to bupivacaine and LE treatment. Cells were loaded with Fluo-3 AM for 30 min, and the intensity was normalized to evaluate changes in Ca^2+^ levels. The bar graphs show the net changes in [Ca^2+^]_i_ and basal Ca^2+^ levels. * *p* < 0.05 compared to each corresponding control (BPV or Fluo). Basal [Ca^2+^]_i_ was measured in the cells incubated with chemicals for 24 h. * *p* < 0.05 compared to the control. ^#^ *p* < 0.05 compared to bupivacaine treatment. (**f** and **g**) Changes in RMP in response to bupivacaine and/or LE in TREK-1 manipulated cells using patch clamping (**f**) and FluoVolt^®^ membrane labeling dye (**g**). * *p* < 0.05 compared to control or each corresponding bupivacaine treatment. ^†^ *p* < 0.05 compared to vector or Sc-siRNA. Each bar represents the mean ± SD of three independent experiments. Arrowheads represent addition of chemicals. FI represents fluorescence intensity (arbitrary units) of cells.

To identify whether bupivacaine and LE affect membrane potentials, plasma and mitochondrial membrane potentials in response to bupivacaine and/or LE were measured with FluoVolt^®^ membrane labeling dye or by whole-cell recording. Bupivacaine depolarized the cells, which showed an increase in the fluorescent intensity with time, whereas combined treatment with LE significantly decreased the fluorescence intensity. Combination of fluoxetine or spadin and LE reduced the fluorescence intensity increased by fluoxetine or spadin (*n* = 36, *p* < 0.05, Figure 6a). As shown in Figure 6a, the depolarized cells showed a contracted shape. Whole-cell recording showed that bupivacaine induced depolarization of resting membrane potential (RMP) to −43.1 ± 3.7 mV from −60.7 ± 4.9 mV (*n* = 6, *p* < 0.05, Figure 6b). The depolarized membrane potential was reduced by a combination of LE and bupivacaine. A combination of fluoxetine or spadin and LE showed a similar effect to bupivacaine and LE (*n* = 6). LE only treatment significantly hyperpolarized the cells (*n* = 14, *p* < 0.05, Figure 6b), but the degree of hyperpolarization was small compared to that in TREK-1 overexpressed cells.

The concentration of potassium ion (K^+^) was measured with a selective K^+^ indicator PBFI-AM. After treatment with bupivacaine, the K^+^ concentration was increased two-fold, whereas the combination of bupivacaine and LE significantly decreased the bupivacaine-induced increase in K^+^ concentration (*n* = 16, *p* < 0.05, Figure 6c). The combined effects of fluoxetine or spadin and LE were similar to combination of bupivacaine and LE (*n* = 12). LE alone treatment significantly decreased the K^+^ concentration (*n* = 16, *p* < 0.05, Figure 6c). MMP, which is measured with JC-1 cationic dye, was also depolarized by bupivacaine treatment. The depolarized H9c2 cells showed high intensity green fluorescence (Figure 6d). The combination of bupivacaine and LE showed lower green fluorescence in the cells compared to bupivacaine only treatment. The red:green fluorescence ratio increased in the combination of bupivacaine and LE (*n* = 18, Figure 6d). The effect of bupivacaine on changes in intracellular Ca^2+^ concentration ([Ca^2+^]_i_), which is related to depolarization of plasma and mitochondrial membrane potentials, was investigated using a calcium indicator (Fluo-3 AM) and a confocal laser scanning microscope. Bupivacaine transiently increased [Ca^2+^]_i_, but the combination of bupivacaine and LE significantly decreased the bupivacaine-induced increase in [Ca^2+^]_i_ (*n* = 18, *p* < 0.05, Figure 6e). LE only treatment slightly increased [Ca^2+^]_i_. LE significantly decreased fluoxetine- or spadin-induced increase in [Ca^2+^]_i_ (*n* = 18, *p* < 0.05). Bupivacaine increased basal [Ca^2+^]_i_, and the combination of bupivacaine and LE significantly decreased the basal [Ca^2+^]_i_ increased by bupivacaine (*n* = 12, *p* < 0.05, Figure 6e).

To identify whether TREK-1 channel expression levels change the effect of bupivacaine and LE on RMP, TREK-1 manipulated cells were adopted. The RMPs of TREK-1 overexpressed cells and TREK-1 knocked-down cells were −80.0 ± 5.3 mV and −49.6 ± 6.9 mV, respectively (*n* = 6, Figure 6f). In TREK-1 overexpressed cells, LE only treatment hyperpolarized RMP. The TREK-1 transfected cells showed a significant decrease in the degree of depolarization by bupivacaine treatment compared to vector transfected cells, whereas TREK-1 siRNA transfected cells showed an insignificant effect of bupivacaine on the membrane potential compared with scrambled siRNA transfected cells. The combination of bupivacaine and LE reduced the depolarization induced by bupivacaine (*n* = 12, *p* < 0.05, Figure 6f).

## 4. Discussion

This study reports a new mechanism whereby TREK-1 channel activation mediates the protective effect of LE on bupivacaine-induced cell death of cardiomyoblasts. TREK-1 background K^+^ channel could be a target for bupivacaine and LE in the heart because bupivacaine inhibits TREK-1 channel activity, and LE activates TREK-1 channel. Blockade of the TREK-1 channel by bupivacaine results in depolarization of RMP and MMP, increase in [Ca^2+^]_i_, and thus the induction of cell death. Activation of TREK-1 channel by LE reduces bupivacaine cytotoxicity through reversal of changes in the membrane potential.

Previous in vitro and in vivo studies demonstrated that LE therapy enhances recovery from cell death and cardiac arrest induced by toxic dose of bupivacaine [7,10,25,26,27,28,29]. Our study also showed a cytotoxicity of bupivacaine and an antagonistic effect of LE on H9c2 cells (Figure 1). The IC_50_ of bupivacaine was approximately 1.2 mM in cell viability tests. The concentration was high compared to that used in other studies. Ok et al. (2016) reported that 300 μM bupivacaine induced 50% cell death of H9c2 [29]. The bupivacaine was obtained from a Pharmaceutical company. Other studies demonstrate that 1 mM bupivacaine induces over 50% cell death [30,31]. The bupivacaine used in other studies was dissolved in different solvents from ours, such as liquid solvent or DMSO. In our study, we used water soluble bupivacaine. Different solvents could affect the permeability of bupivacaine. Although the in vitro concentration highly exceeds the blood concentration of bupivacaine, revealing the mechanism of bupivacaine toxicity and LE’s reversal effect is important for understanding their actions and use in clinic. In the human body, the bupivacaine could work at low concentrations through the identified mechanism. The human body will be more sensitive to most chemicals than cell line, because it is systemically and finely regulated by a variety of factors.

TREK-1 expression level is affected by bupivacaine and/or LE (Figure 2). Numerous types of Na^+^, Ca^2+^, and K^+^ channels expressed in the cardiovascular cells have been suggested as therapeutic targets for cardiac diseases, because they play important roles in cardiac physiology, such as generation of action potential [32,33]. Cardiac K^+^ channels, which are the targets for the action of antiarrhythmic drugs, are divided into three broad types: voltage-gated (K_v_), inward rectifier (K_ir_), and background K^+^ (K_2P_) channels [32]. TREK-1 channel belongs to the background K^+^ channels (K_2P_), which set the resting membrane potential. TREK-1 is a member of the mechanosensitive K_2P_ subfamily, which includes TREK-2 and TRAAK channels. They are activated by application of stretch, polyunsaturated fatty acids (PUFAs), and intracellular acidification [14]. However, in H9c2 cells, we could not detect TREK-2 and TRAAK mRNAs despite several attempts. Therefore, we focused on the TREK-1 channel in H9c2 cells. The TREK-1 channels are expressed in cardiac organs from Drosophila to humans, and regulate cardiac fibrosis, diastolic dysfunction, and sinoatrial node membrane excitability [12,34]. Our finding is in line with the studies that confirmed TREK-1′s role in the cardiovascular system. Bupivacaine showed a low toxicity in the TREK-1 overexpressed H9c2 cells, and a high toxicity in TREK-1 knocked-down cells (Figure 3). In addition, the expression level of the TREK-1 channel alone affected the cell viability. TREK-1 overexpressed and knocked-down cells showed an increase and a decrease in cell viability, respectively. These results indicate that TREK-1 channel has a potential protective effect on cytotoxicity induced by high concentrations of bupivacaine. Although bupivacaine reduces the TREK-1 expression, the inhibitory effect of bupivacaine on TREK-1 expression is not 100%, so some of the TREK-1-expressing cells may survive from bupivacaine toxicity. High TREK-1-expressing cells might be protected from bupivacaine toxicity by the single action of TREK-1 or TREK-1 activation by LE. However, there is a gap between the increase in the channel expression and the channel activity. Channel activation can be immediately induced by chemicals, but changes in channel expression require time.

In addition to expression level, TREK-1 channel activity is modulated by bupivacaine and LE. TREK-1 channel activity is more sensitive to bupivacaine than LE with an IC_50_ of approximately 300 μM. Faster TREK-1 activation could help reduce bupivacaine toxicity. TREK-1 channel modulators regulate cell viability of H9c2 cells, indicating that the TREK-1 channel is functionally expressed in H9c2 cells (Figure 4 and Figure 5). The pharmacological responses to bupivacaine and LE between the heterologous TREK-1 channel and native TREK-1 channel were almost identical, but we observed a difference in the degree of the response to LE only treatment between them. The small amount of activation by LE in H9c2 compared to TREK-1 overexpressed cells could result from other K^+^ channels expressed in H9c2 cells. The difference could be due to K^+^ channels inhibited by LE (PUFA) among K^+^ channels unaffected by 1 mM 4-AP, 1 mM Ba^2+^, and 1 mM TEA [35]. However, the low activation of TREK-1 channel in H9c2 cells could make a significant effect on membrane potential. LE alone treatment significantly hyperpolarized the membrane potential by approximately 10 mV (Figure 6). A 10–20 mV change in RMP in H9c2 cells is relatively large, and the change could have a strong effect on cell electrical activity and cell function. Spadin-induced depolarization of RMP by approximately 12 mV potentiated insulin secretion in Min6-b1 cells [36]. In addition, the low activation of TREK-1 currents by LE in H9c2 cells induced a K^+^ exit and a decrease of intracellular K^+^ concentration ([K^+^]_i_). The change in [K^+^]_i_ was significant, but the degree of change was small (control, 64.7 ± 8.8 vs. LE, 55.2 ± 10.9). These results indicate that TREK-1 is a functional background K^+^ channel with a big unitary conductance in H9c2 cells. TREK-1 channel is highly active at body temperature compare to room temperature. These functions will be higher in in vivo. In the combined effect of other TREK-1 channel inhibitors and LE, combination of fluoxetine or spadin and LE showed a similar effect to the combination of bupivacaine and LE.

Generally, TREK-1 activation induces hyperpolarization of the membrane potential, and the change in membrane potential is a rapid action in response to ion channel modulators and regulatory factors. Changes in PMP are an important early event in cell death [37]. The depolarization of the PMP induces increase in [Ca^2+^]_i_, and the increased [Ca^2+^]_i_ elicits a loss in MMP [38]. [Ca^2+^]_i_ increased by bupivacaine induced contraction of H9c2 cells (Figure 6). These results indicate that modulation of the TREK-1 channel might be involved in the cell death process. The TREK-1 channel, a stretch-activated channel, has been implicated as a protective channel in normal and pathogenic cardiovascular conditions based on its pharmacological properties [13,32,39]. TREK-1 channels modulate cardiovascular response to membrane stretch, PUFAs, extracellular and intracellular pH, and temperature. Our study adds cardioprotective effects to the existing TREK-1 effects database. The TREK-1 channel might be used as a drug target (e.g., bupivacaine, LE, and so on) for the treatment of cardiovascular disorders, and its activators could be used as a cardioprotective medication.

However, this study has some limitations. Based on our experimental results, we suggest that TREK-1 channel plays an important role in the viability of cardiac cells. However, the cell viability rate was statistically significant before and after TREK-1 manipulation, but the level of change was not high. These results can raise questions about biological value. However, the numerical values could be different among experimental methods analyzing cell death. Lactate dehydrogenase (LDH)-based assay commonly uses for analysis of cytotoxicity. The numerical values obtained from LDH-based assay were approximately 30% higher than those from MTT-based assay (data not shown). In addition, TREK-1 manipulated cells showed bigger change in membrane potential than that in chemical (bupivacaine and LE) treated cells. Chemical treatments can simultaneously and sequentially control various molecules that are affected by the chemical. Bupivacaine treatment can block cation channels including Na^+^ channel, thus reducing the degree of depolarization. The difference in gene expression and chemical treatment in the membrane potential can raise the question that TREK-1 may not be the primary target of bupivacaine and LE. However, bupivacaine and LE significantly regulated cell viability in the presence of a strong Na^+^ channel blocker through changes in membrane potential and Ca^2+^ signals. Further studies are needed to identify the functional expression of the TREK-1 channel, to understand the modulators binding sites, to investigate the death property of bupivacaine and TREK-1 knockdown, and to find molecules that interact with the bupivacaine and LE in primary human cardiovascular cells. Other K^+^ channels expressed in human heart cells should be studied together with TREK-1 to precisely identify the drugs effect.

## 5. Conclusions

Taken together, this study demonstrates that TREK-1 channel is involved in the bupivacaine cytotoxicity and mediates an antagonistic effect of LE on bupivacaine-induced cytotoxicity of H9c2 cells. TREK-1 activation reversed bupivacaine-induced depolarization of membrane potential. These results indicate that the TREK-1 channel mediates setting the resting membrane potentials as a background K^+^ channel in H9c2 cells and could be a target for the action of bupivacaine and LE.

## Figures and Tables

**Table 1:** Primer sequences used for reverse transcriptase-PCR and real-time PCR.

Gene Name (Channel Name)	GenBank Accession No.	Primer Sequences (5′–3′)	Expected Size (bp)	Application
*KCNK2*	AF385402	Sense: TCTGAATGAATCAGAATGCTTTGCTA Antisense: TCATTCCTAAGTGGTCAGGTATCCAG	106	Real-time PCR
(TREK-1)		Sense: TGCCAAAGTGGAGGACACAT Antisense: CTCTCCCACCTCTTCCTTCG	361	RT-PCR
*KCNK10* (TREK-2)	AF196965	Sense: CAGCCCAAGAGTGCCACTAA Antisense: GGATCCCAAAGATGGCGTAT	493	RT-PCR
*KCNK4* (TRAAK)	AF302842	Sense: CACCACTGTAGGCTTTGGCGATTATG Antisense: ACTCTGCGTGTCTGAGGACTCGTCG	445	RT-PCR
*GAPDH*	NM_017008	Sense: CATGGCCTTCCGTGTTC Antisense: CTGCTTCACCACCTTCTT	103	Real-time PCR
		Sense: CTAAAGGGCATCCTGGGC Antisense: TTACTCCTTGGAGGCCATG	201	RT-PCR
*RPS12*	M18547	Sense: TGAGCCCATGTATGTCAAGCTGGT Antisense: ACTACAACGCAACTGCAACCAACC	162	Real-time PCR
